# Extracellular Vesicles in Breast Cancer: From Intercellular Communication to Therapeutic Opportunities

**DOI:** 10.3390/pharmaceutics16050654

**Published:** 2024-05-14

**Authors:** Barathan Muttiah, Sook Luan Ng, Yogeswaran Lokanathan, Min Hwei Ng, Jia Xian Law

**Affiliations:** 1 Centre for Tissue Engineering and Regenerative Medicine, Faculty of Medicine, Universiti Kebangsaan Malaysia, Cheras, Kuala Lumpur 56000, Malaysia; lyoges@ppukm.ukm.edu.my (Y.L.); angela@ukm.edu.my (M.H.N.); 2 Department of Craniofacial Diagnostics and Biosciences, Faculty of Dentistry, Universiti Kebangsaan Malaysia, Jalan Raja Muda Abdul Aziz, Kuala Lumpur 50300, Malaysia; ngsookluan@ukm.edu.my

**Keywords:** breast cancer, drug delivery, extracellular vesicles, nanotechnology, therapeutic resistance

## Abstract

Breast cancer, a multifaceted and heterogeneous disease, poses significant challenges in terms of understanding its intricate resistance mechanisms and devising effective therapeutic strategies. This review provides a comprehensive overview of the intricate landscape of extracellular vesicles (EVs) in the context of breast cancer, highlighting their diverse subtypes, biogenesis, and roles in intercellular communication within the tumour microenvironment (TME). The discussion spans various aspects, from EVs and stromal cells in breast cancer to their influence on angiogenesis, immune response, and chemoresistance. The impact of EV production in different culture systems, including two dimensional (2D), three dimensional (3D), and organoid models, is explored. Furthermore, this review delves into the therapeutic potential of EVs in breast cancer, presenting emerging strategies such as engineered EVs for gene delivery, nanoplatforms for targeted chemotherapy, and disrupting tumour derived EVs as a treatment approach. Understanding these complex interactions of EV within the breast cancer milieu is crucial for identifying resistance mechanisms and developing new therapeutic targets.

## 1. Introduction

Breast cancer is a pervasive global health challenge, ranking as the most common cancer among women worldwide [1]. Incidence rates vary across regions, with higher occurrences in developed countries due to extensive mammography screening, and transitioning countries such as Iran, China, and Mexico have comparatively lower rates. Breast cancer remains a leading cause of cancer-related deaths. Factors such as age, genetics, hormonal influences, and lifestyle choices contribute to its prevalence [2]. As of 2020, breast cancer remains a global health challenge, with 2.3 million women diagnosed and 685,000 deaths reported [3]. Asia accounted for nearly half (45.4%) of the 2.3 million cases of breast cancer that were diagnosed in 2020 [4]. In 2050, there will be 1,503,694 breast cancer death cases worldwide (1,481,463 women and 22,231 men) [5]. The peak age of breast cancer onset in some Asian countries is notably younger than in Europe and North America. The decline in breast cancer incidence in the USA and the United Kingdom, particularly in women aged 50–59 is probably linked to decreased use of hormonal treatment [6]. However, rising incidence rates in China and South Korea reflect changing reproductive patterns and lifestyle factors associated with economic development [7]. Meanwhile, breast cancer is the overall most common cancer type in Malaysia [8]. There has been a projected 31% increase in new breast cancer cases globally by 2040 [5], highlighting the need for continued efforts in prevention, early detection, and treatment to address this growing public health challenge. Despite being the most prevalent cancer worldwide, the 5-year relative survival rate for women with invasive breast cancer is 86% [9], which is a noteworthy advancement in breast cancer survival compared to Malaysia’s 71.4–74.9% [10]. By the end of 2020, 7.8 million women who had been diagnosed with breast cancer in the past five years were alive, reflecting the impact of improved detection and treatment programs globally [11].

Breast cancer encompasses a spectrum of diseases, including non-invasive and invasive types [12]. Non-invasive breast cancers, such as ductal carcinoma in situ (DCIS) and lobular carcinoma in situ (LCIS), originate within breast tissue without invading surrounding areas, posing a lower immediate threat but requiring monitoring as they can progress to invasive cancer [13]. Invasive breast cancers, predominantly invasive ductal carcinoma (IDC) and invasive lobular carcinoma (ILC), breach the ducts or lobules, spreading to nearby tissues and potentially metastasizing to distant organs [14]. These invasive cancers are further classified based on molecular characteristics, such as hormone receptor status (HR-positive or HER2-positive) and triple-negative status (lacking expression of oestrogen, progesterone, and HER2 receptors), influencing treatment strategies and prognosis. HR-positive tumours respond to hormone therapies, while HER2-positive cancers benefit from targeted HER2 inhibitors [15]. Triple-negative breast cancer (TNBC) presents challenges due to the absence of these targets, requiring aggressive treatments like chemotherapy and radiation, alongside emerging targeted therapies under investigation. Ongoing research aims to improve outcomes across all breast cancer subtypes through better understanding and tailored therapeutic approaches [16]. The ongoing efforts in early detection, treatment advancements, and global initiatives continue to shape the landscape of breast cancer management and contribute to improved outcomes for affected individuals [17].

The challenges in breast cancer treatment, including tumour heterogeneity and drug resistance, highlight the need for newer drugs. Understanding hypoxia, autophagy, apoptosis, and the tumour microenvironment (TME) is crucial for overcoming resistance and developing effective therapies [18]. EVs play a key role in breast cancer by promoting angiogenesis, immune modulation, and pre-metastatic niche formation [19]. Investigating extracellular vesicles’s (EVs) involvement in hypoxia, autophagy, apoptosis, and TME modulation is essential. Addressing these complexities through research and development efforts can enhance breast cancer care and patient outcomes, contributing to oncology advancements.

## 2. A Complex Interplay

### 2.1. Hypoxia

In animals under limiting oxygen conditions, the expression of a large set of genes is transcriptionally activated at the cellular level to form part of the oxygen homeostasis and adaptive response [20]. The hypoxia-inducible factors (HIFs) are the key transcription factors mediating this cellular transcriptional response. Prolyl hydroxylation of HIFα is catalysed by the HIF prolyl-hydroxylases (PHD1–3 in human), leading to HIFα degradation via the polyubiquitination by VHL complex [21]. The HIF asparaginyl hydroxylase, factor inhibiting HIF (FIH), catalyses the oxygen-dependent hydroxylation of HIFα which blocks HIFα association with co-activators p300/CBP, thereby reducing HIF transcriptional activity [22]. Under limiting oxygen concentrations, the activity of both the PHDs and FIH are suppressed, leading to the stabilisation and activation of the HIF. Upregulation of HIFα is frequently observed in solid tumours and has been linked to poor patient prognosis [23]. Furthermore, solid tumour microenvironment is often hypoxic due to the high rate of cell proliferation and abnormal vasculature. For example, in breast cancer, both isoforms of HIFα (HIF1α and HIF2α) are linked to aggressive phenotype and poor prognosis, respectively [24]. However, a previous study (Chen et al. explored) using two distinct subtypes of human breast cancer cell lines cultured in two dimensional (2D) and hypoxia-mimicking agents (HMAs) were used to induce hypoxia. The finding has showed opposing proliferative effects against one breast cancer cell type upon pharmacological activation of HIF. Therefore, the role of the hypoxia pathway (and thus, HIF) is highly complex in cancer and warrants further studies, especially in an advanced and more physiologically-relevant cellular cancer model such as the PDOs [25]. Studying the molecular responses, particularly the activation of HIFs, provides insights into the signalling pathways that contribute to breast cancer progression and resistance.

### 2.2. Autophagy

Autophagy, a complex catabolic process, plays a crucial role in maintaining cellular homeostasis by forming double-membrane vesicles known as autophagosomes [26]. These autophagosomes engulf and transport intracellular contents to lysosomes for degradation by hydrolytic enzymes, providing energy and building blocks for cellular recycling [27]. The proper functioning of autophagy is essential for cancer prevention, as it contributes to the clearance of damaged organelles and aggregated molecules in normal cells. Dysfunctional autophagy can significantly impact cell fate and contribute to tumourigenesis [28]. Increased autophagy-related gene signatures are observed in normal mammary glands, which are lost during breast cancer progression. Notably, the autophagy-related gene Beclin1 has been linked to breast cancer, with Beclin1 deficiency associated with tumourigenesis [29]. Conversely, increased Beclin1 activity has been shown to prevent the progression of HER2-positive tumours [30]. The tumour-suppressive effects of autophagy in breast cancer are primarily attributed to Beclin1, frequently found to be monoallelically deleted in human breast cancer cells [31]. The tumour-suppressive Forkhead Box O (FOXO) transcription factors, responsible for regulating cellular homeostasis, stem cell maintenance, and aging, are associated with promoting autophagy. The loss of FOXO3 reduces autophagic activity and induces mammary tumour formation [32]. Identifying specific autophagy-related genes and pathways involved in breast cancer allows for the exploration of potential therapeutic targets to enhance treatment efficacy.

### 2.3. Apoptosis

Dysregulation of apoptosis, the programmed cell death process, is a common feature in cancer, including breast cancer [33]. Apoptosis is a tightly regulated mechanism that eliminates damaged or unwanted cells, playing a crucial role in maintaining tissue homeostasis. In cancer, this balance is disrupted, and cancer cells often acquire the ability to evade apoptosis, contributing to uncontrolled cell growth and tumour progression. In breast cancer, dysregulation of apoptosis can occur through various mechanisms, including alterations in the expression or function of key proteins involved in the apoptotic pathway [34]. Some factors contributing to apoptosis dysregulation in breast cancer include p53 mutation, over expression of HER2, activation of the PI3K/AKT/mTOR pathway and overexpression of anti-apoptotic proteins such as r B-cell leukemia/lymphoma 2 (Bcl-2) [35,36]. Identifying factors contributing to apoptosis dysregulation, such as p53 mutations and altered expression of apoptosis-related proteins, offers opportunities for therapeutic intervention to restore normal cell death mechanisms.

### 2.4. Tumour Microenvironment (TME)

TME in breast cancer is a complex ecosystem comprising various cell types, including immune cells, tumour-associated fibroblasts, endothelial cells, and extracellular matrix components (ECM) [37]. These components interact dynamically, shaping tumour behaviour and therapeutic responses. Immune cells within the TME, such as tumour-infiltrating lymphocytes (TILs) and tumour-associated macrophages (TAMs), play crucial roles in tumour progression and treatment outcomes [38]. TILs, including CD8^+^ cytotoxic T cells and CD4^+^ helper T cells, have prognostic and predictive value across different breast cancer subtypes. TAMs, which can adopt pro-tumorigenic M2 phenotypes, contribute to tumour growth and therapy resistance [39]. Cancer-associated fibroblasts (CAFs) are key stromal cells that regulate tumour initiation, angiogenesis, and metastasis through extracellular matrix remodelling and secretion of growth factors. Angiogenesis, facilitated by factors like hypoxia-inducible factor (HIF), supports tumour growth and metastasis by promoting the formation of new blood vessels [40]. Additionally, dysfunctional adipocytes in the TME, known as cancer-associated adipocytes (CAAs), contribute to breast cancer progression through secretion of pro-tumorigenic factors. Understanding these interactions within the TME is essential for developing targeted therapies and prognostic markers to improve outcomes for breast cancer patients [41]. The Hedgehog (Hh), Wnt, Notch, and NF-κB pathways not only influence metastasis individually but also cross-regulate each other, further increasing TME complexity and fuelling cancer progression. The Hh and Notch signalling pathways are involved in mediating interactions between cancer cells and macrophages, contributing to the dynamics within the TME [42]. Notch facilitates communication between cancer cells and TME components; it can act as either an oncogene or a tumour suppressor. Meanwhile, the Hedgehog (Hh) pathway also plays a critical role in mediating interactions between cancer cells and the TME. Activation of the Hh pathway has been associated with the recruitment of stromal cells, including macrophages, to the TME. Wnt is involved in controlling factors responsible for epithelial-mesenchymal transition (EMT), a key step in metastasis [43]. Lastly, NF-κB regulates genes that control the inflammatory response, promoting tumour growth. This dynamic interplay between cancer cells and the TME significantly influences metastatic potential. Stromal cells, immune components, mesenchymal stem cells, and myeloid-derived suppressor cells contribute to the cellular milieu, while the ECM provides structural support [44]. Signalling molecules, including cytokines, chemokines, and growth factors, mediate communication within the TME. Abnormal blood vessels, hypoxia, and immune responses play pivotal roles. The TME’s reciprocal influence on cancer cells and vice versa shapes tumour progression, invasion, and metastasis [45]. Since signalling pathways like Hh, Wnt, Notch, and NF-κB within the TME influence metastasis and cancer progression, identifying specific targets within these pathways may offer therapeutic opportunities to disrupt the supportive microenvironment and impede metastatic spread [46]. The understanding of the TME has evolved significantly since Stephen Paget’s pioneering “seed and soil” theory proposed in 1889 [47].

Traditionally, cancer research has primarily focused on intrinsic factors of cancer cells, but in recent years, there has been a paradigm shift towards recognizing the crucial role of the TME in cancer development and progression [48]. This shift has led to the exploration of novel therapeutic strategies targeting the TME, particularly in breast cancer. Immune checkpoint inhibitors (ICIs), such as programmed cell death protein 1 (PD-1), programmed cell death protein ligand-1 (PD-L1), and cytotoxic T-lymphocyte-associated protein 4 (CTLA-4) inhibitors, have emerged as promising treatments, especially in TNBC, where increased immune cell infiltration makes it more susceptible to immunotherapy. Clinical trials combining ICIs with chemotherapy have shown significant improvements in overall survival and response rates among patients with advanced non-small cell lung cancer (NSCLC) [49]. Another randomized controlled trial, encompassing 4468 patients, evaluated PD-1/PD-L1 inhibitors combined with chemotherapy in triple-negative breast cancer (TNBC) patients. The findings indicate significant improvements in pathological complete response (pCR), event-free survival (EFS), and overall survival (OS) compared to chemotherapy alone or placebo plus chemotherapy [50]. Additionally, strategies to modulate TAMs for therapeutic purposes, such as bisphosphonates such as oral bisphosphonate clodronate, zoledronic acid and pamidronate, are commonly used in the treatment of bone metastases in various cancers, including breast cancer. Their ability to modulate TAMs may contribute to their anti-tumour effects beyond their role in bone protection, in addition, antibody-drug conjugates (ADCs) targeting antigens such as CD47, CD163, and CSF1R are being investigated [51]. In hormone receptor-positive breast cancer, targeting the TME with aromatase inhibitors (AIs) proves highly effective. Both steroidal (e.g., exemestane) and non-steroidal (e.g., anastrozole and letrozole) demonstrate statistically equivalent effectiveness in long-term treatment. Notably, studies haven’t shown a significant advantage for 5 years of continuous AI therapy compared to a sequence of 2 years of tamoxifen followed by 3 years of AI treatment [52]. Meanwhile, anti-angiogenic agents like bevacizumab, has demonstrated efficacy in both adjuvant and metastatic settings whereby in a phase 3 trial for operable triple-negative breast cancer, patients received chemotherapy alone or with bevacizumab [53]. After about 31.5 months, no significant survival difference was observed between the groups. Bevacizumab showed potential benefit in patients with high pre-treatment VEGFR-2 levels but increased adverse events like hypertension. The study suggests caution in recommending bevacizumab for adjuvant treatment pending further survival analysis [54]. Furthermore, emerging research is focusing on the role of CAAs and their secretome in promoting tumour progression, suggesting new avenues for therapeutic intervention. Overall, harnessing the complex interactions within the TME offers promising opportunities for developing more effective and personalized treatments for breast cancer patients.

### 2.5. Cancer Stem Cells (CSCs) 

The breast CSC (BCSC) niche consists of various non-malignant cells that interact with CSCs, promoting self-renewal, therapy resistance, and metastasis [55]. BCSCs exhibit plasticity and can transition between different states, such as mesenchymal-like and epithelial-like phenotypes [56]. These phenotypic variations contribute to tumour heterogeneity and metastatic potential. High levels of CSCs within tumours are associated with poor prognosis and increased risk of cancer relapse [57]. However, CSCs also exhibit resistance to conventional cancer therapies, including chemotherapy and radiation [58]. They activate molecular pathways that enhance DNA repair mechanisms, detoxification enzymes, and anti-oxidant capabilities, rendering them resistant to treatment. One such mechanism involves the downregulation of major histocompatibility complex class I (MHC-I) molecules and molecules involved in peptide loading, rendering BCSCs less recognizable by CD8^+^ T cells [59]. However, the absence of MHC-I may render BCSCs susceptible to killing by Natural Killer (NK) cells, which preferentially target BCSCs due to the upregulation of NK ligands [60]. Additionally, radiotherapy can increase the expression of stress ligands on surviving BCSCs, enhancing their susceptibility to NK cell-mediated killing. Despite this, BCSCs may exhibit reduced susceptibility to NK-mediated killing due to the downregulation of NK-activating NKG2D ligands, allowing them to escape immune surveillance [61]. Furthermore, BCSCs overexpress several immunosuppressive molecules, including immune checkpoint (IC) ligands such as (PD-L1), PD-L2, CD276, CD155, CD200, and CD47. These molecules weaken the activity of NK and T cells and promote stemness in BCSCs. For example, PD-L1 and PD-L2 inhibit NK and effector T-cell function while promoting stemness by inducing the expression of stem cell markers. Similarly, CD276, CD155, CD200, and CD47 inhibit T and NK cell killing and induce an immunosuppressive tumour microenvironment [62,63]. In addition to immune checkpoint ligands, BCSCs secrete immunosuppressive cytokines and molecules such as transforming growth factor (TGF)-β, interleukin (IL)-6, IL-8, and vascular endothelial growth factor (VEGF) [64,65]. These factors convert immune cells into tumour-promoting allies and contribute to tumour progression and metastasis. For instance, TGF-β induces epithelial-to-mesenchymal transition (EMT), promotes angiogenesis, and induces immunosuppression by favouring the infiltration of T regulatory cells (Tregs) and myeloid-derived suppressor cells (MDSCs) into tumours [66]. IL-6 and IL-8 further sustain BCSCs’ stem-like qualities and progression by stimulating the expression of genes involved in stemness, tumorigenesis, migration, and metastasis [67]. 

### 2.6. Other Apoptosis Resistant in Breast Cancer 

Breast cancer cells often develop resistance to apoptosis, allowing them to evade cell death and proliferate uncontrollably [68]. This resistance is attributed to various factors, including upregulation of antiapoptotic proteins like BCL-2 family members. Genetic changes and dysregulation of gene expression can disrupt the balance between cell proliferation and apoptosis, contributing to malignant transformation and tumour growth [69]. Mutations or deletions of the p53 gene are found in a significant portion of breast cancer tissues, and this alteration is associated with a poor prognosis for patients [70]. Wild-type p53 protein, when expressed normally, induces apoptosis in tumour cells, regardless of p53 mutation status. It does so by activating the expression of several proapoptotic genes, including Fas, TNF-related apoptosis-inducing ligand (TRAIL) receptors, and Bcl-2-associated X-protein (BAX) [71]. Thus, mutations in p53 or downstream deficiencies in the p53 pathway can lead to resistance to apoptosis in breast cancer cells [68]. Additionally, the interaction between p53 and apoptosis-stimulating proteins (ASPP) enhances p53’s ability to induce apoptosis, but ASPP is often downregulated in breast cancer tissues, reducing the sensitivity of cancer cells to apoptosis stimuli [72]. Meanwhile, in a significant proportion of breast cancers, there is overexpression or amplification of tyrosine kinase receptors such as epidermal growth factor receptor (EGFR) and HER2, leading to increased activation of the phosphoinositide 3-kinases (PI3K) pathway [73]. This pathway promotes cell survival, proliferation, metastasis, and angiogenesis while inhibiting apoptosis. PI3K activates AKT, which then phosphorylates and inhibits proapoptotic factors such as Bcl-2-associated death promoter (BAD) and caspase 9, decreases p53 levels, and reduces the expression of proapoptotic factors like Fas-L [74]. Additionally, AKT activates factors that promote cell proliferation. The upregulation of PI3K/AKT signalling in breast cancer tissues decreases the sensitivity of these cells to apoptosis induction. However, the tumour suppressor gene phosphatase and tensin homolog (PTEN) acts as a negative regulator of the PI3K pathway by antagonizing its signalling [75]. PTEN dephosphorylates a lipid product required for AKT activation, thus inhibiting the PI3K pathway. Deletion or mutation of PTEN is found in a significant portion of breast cancers and is associated with reduced apoptosis and increased tumour development in animal models [76]. Inhibitor of apoptosis (IAP) family proteins, including X-linked IAP (XIAP) and survivin, play critical roles in apoptosis resistance. Upregulation of XIAP inhibitscaspase activity and promotes resistance to apoptosis induced by various stimuli, including chemotherapy and radiotherapy [77]. Survivin, which is normally absent in adult tissues but highly expressed in tumours, contributes to a higher apoptotic threshold in breast tumour cells. Downregulation of the proapoptotic protein XIAP-associated factor 1 (XAF1), which counteracts XIAP inhibition, is observed in breast cancer cell lines and tissues [78]. 

### 2.7. Angiogenesis

Angiogenesis, the process of new blood vessel formation, is critical for tumour growth and metastasis in breast cancer [79]. HIFs play a central role in regulating angiogenesis by controlling the expression of genes involved in metabolism, angiogenesis, and cell division. Tumour angiogenesis is initiated when pro-angiogenic factors such as VEGF and fibroblast growth factors (FGFs) disrupt the balance between pro- and anti-angiogenic factors in the tumour microenvironment [80]. This process leads to the recruitment of endothelial cells (ECs) and the formation of new blood vessels, supplying the tumour with oxygen and nutrients. The dysregulation of angiogenesis is a hallmark of cancer progression, and elevated levels of angiogenic factors are associated with poor prognosis in breast cancer patients [81]. In breast cancer, angiogenesis is essential for tumour growth, progression, and metastasis. Tumour cells produce angiogenic factors such as VEGF interleukin-8 (IL-8), basic fibroblast growth factor (bFGF/FGF-2), and matrix metalloproteinases (MMPs), which promote the formation of new blood vessels [82]. Elevated levels of angiogenic growth factors correlate with the aggressiveness and risk of invasive breast cancer and are associated with poor prognosis. The interaction between VEGF and its receptors, particularly VEGFR-1 and VEGFR-2, plays a crucial role in breast cancer development, progression, and metastasis [83]. Additionally, IL-8 enhances angiogenesis by stimulating the production of VEGF in endothelial cells (ECs) and promoting EC proliferation, survival, and migration. bFGF/FGF-2 and MMPs also contribute to angiogenesis and tumour progression by modulating extracellular matrix remodelling and destabilizing existing blood vessel walls. Targeting these pro-angiogenic factors and their receptors has been explored as a therapeutic strategy for breast cancer and other angiogenic diseases [84]. Research delves into the role of circular RNAs (circRNAs) in contributing to various aspects of breast carcinogenesis, including angiogenesis, cell proliferation, apoptosis, epitheli-al-to-mesenchymal transition (EMT), metastasis, and drug resistance. Nearly ten circRNAs, including hsa_circ_0005046, hsa_circ_0001791, hsa_circ_006054, hsa_circ_100219, circVRK1, circAGFG1, circSEPT9, circTADA2A-E6, circTADA2A-E5/E6, and hsa_circ_0044234, have demonstrated a high potential as angiogenesis marker [85]. Table 1 demonstrated the overall cellular mechanisms of breast cancer progression. 

By illuminating the involvement of EVs in hypoxia, autophagy, apoptosis, and modulation of the TME, we can unravel the complexities of breast cancer biology [86]. Tackling these intricacies through dedicated research and development endeavours is imperative for propelling breast cancer care forward, enhancing patient outcomes, and driving continued advancements in the field of oncology.

## 3. Extracellular Vesicles

EVs are membrane-derived vesicles of varying sizes, ranging from 40 to 1000 nm, continuously released by both eukaryotic and prokaryotic cells into biological fluids [87]. EVs were initially described by Wolf in 1967 as “platelet dust” produced during platelet separation from plasma, later termed EVs [88]. Clancy et al. observed EV release from both normal and tumour cells, implicating them in pathophysiological processes [89]. Salomon et al. identified reticulocyte-released vesicles during maturation, termed “exosomes”. EVs are classified into subtypes, including exomeres, exosomes, microvesicles, apoptotic bodies, migrasomes, and oncosomes [90]. The International Society for Extracellular Vesicles recommends the generic term “EVs” due to subtype marker absence. EVs are classified based on size (“small EVs” (sEVs) and “medium/large EVs” (m/lEVs) with defined size ranges) or density (low, middle, high), origin (e.g., podocyte EVs, hypoxic EVs, large oncosomes, apoptotic bodies), biochemical components (e.g., CD63^+^/CD81^+^-EVs, Annexin A5-stained EVs), and even the physiological state of the producing cell [91]. Hence, operational terms such as “small EVs” and “large EVs” are encouraged to describe subpopulations of EVs based on characteristics such as size, density, molecular composition, or cellular origin [92].

Tumour-derived EVs carry cargo that can influence recipient cells, potentially promoting tumour cell migration, invasion, angiogenesis, and immune evasion, contributing to cancer progression [93]. Exosomes, well-studied nanovesicles, are formed through complex endocytic pathways involving early endosome formation, intraluminal vesicle (ILV) generation, and multivesicular body (MVB) fusion with the plasma membrane [94]. These exosomes typically range in size from 30 to 150 nm. Meanwhile, exomeres, non-membrane vesicles smaller than 50 nm nanoparticles, were recently discovered, lacking lipid bilayers. Microvesicles (MVs), larger in size (100–1000 nm), are released directly through the plasma membrane. MVs directly bud from the cell membrane and are promoted by the endocytic–lysosomal (ESCRT) pathway, increased Ca^2+^, and cytoskeleton changes [95]. Migrasomes, migration-dependent membrane-bound vesicular structures that contain cellular contents and small vesicles, is typically in the range of 500–3000 nm [96]. Apoptotic bodies, varying in size from 1 to 5 μm, are heterogeneous vesicles released during programmed cell death and encapsulate dying cell contents [97]. Lastly, large oncosomes typically in the range of 1–10 µm, carriers of carcinogens, are larger vesicles released by tumour cells through amoeba-like movement [98]. Despite their heterogeneity, each EV possesses unique characteristics, contributing to the complexity of accurately differentiating subtypes. Understanding EVs, their biogenesis, and role in intercellular communication provides insights into physiological and pathological processes, with potential implications for therapeutic development [99]. Table 2 described the common EV subtypes and their general characteristics. 

### 3.1. EVs and Stromal Cells in Breast Cancer

Within the stroma, various cell types play crucial roles in the dynamics of tumour-stromal interactions. Key players include cancer-associated fibroblasts, endothelial cells, immune cells, and mesenchymal stromal cells (MSCs) [100]. Components expressed by the stroma of breast carcinomas, such as matrix metalloproteases, their tissue inhibitors, integrins, cytokines, or toll-like receptors, are associated with metastasis development [101]. Notably, based on the expression of these factors, two distinct types of breast cancer stroma can be identified, exerting significantly different influences on patient prognosis. Evidence also suggests the existence of bidirectional signalling between cancer cells and tumour stroma cells, with prognostic implications [102]. This insight opens up avenues for novel therapeutic strategies in breast cancer. Understanding the complex interactions within the tumour microenvironment, including the stromal components, is crucial for developing targeted and effective treatments to improve patient outcomes since the intercellular communication between cancer cells and the TME is mediated, in part, by EVs. Recently, single-cell RNA sequencing of five triple-negative breast cancer unveiled two distinct subpopulations of CAFs and perivascular-like (PVL) cells [103]. CAFs exhibited two states—one resembling myofibroblasts and the other characterized by high expression of growth factors and immunomodulatory molecules [104]. PVL cells clustered into two states consistent with a differentiated and immature phenotype. These stromal states exhibited distinct morphologies, spatial relationships, and functional properties in regulating the extracellular matrix [103]. A study has found that the intricate crosstalk orchestrated by stromal cells through exosomes which carried RNA and signalling pathways enhances the resistance of breast cancer cells to therapy [105]. Researchers investigated TNBC-derived exosomes and their cargo of microRNAs (miRNAs), including miR-185-5p, miR-652-5p, and miR-1246, in the activation of normal fibroblasts (NFs) into CAFs, enhanced the invasion potential of normal breast epithelial cells, resembling the in vivo TME [106]. Another specific example is miR-105, which is secreted by breast cancer cells in EVs and has been implicated in mediating metabolic reprogramming of CAFs through MYC signalling and contributes to breast cancer growth [107]. A study that delves into the intricate role of EVs containing miR-205 and miR-31 derived from human MSCs in breast cancer metastasis found that these EVs supported the progression of the primary breast tumour and they also concurrently suppressed metastasis in breast cancer cells that lacked organ-specific commitment [108]. Inhibition of these miRNAs could potentially prevent metastasis or even induce dormancy in cancer cells.

### 3.2. Breast Cancer-Secreted EVs Targeting Angiogenesis

Angiogenesis, the formation of new blood vessels, plays a crucial role in tumour growth and metastasis across various cancers, including breast cancer [109]. Stromal Interaction Molecule 1 (STIM1) promotes angiogenesis by reducing exosomal miR-145 in breast cancer MDA-MB-231 cells. The findings shed light on the complex interplay between STIM1, exosomal miR-145, and angiogenesis, offering potential therapeutic insights [110]. Exosomal Small Nucleolar RNA Host Gene 1 (SNHG1) from hypoxic breast cancer cells could enhance tumour angiogenesis and growth by regulating the miR-216b-5p/JAK2 axis [111]. The findings suggest that SNHG1 may serve as a potential therapeutic target for breast cancer. Interestingly, serum exosomal-annexin A2 (exo-AnxA2) has been linked to angiogenesis and metastasis in breast cancer. The study has found that higher levels of exosomal-AnxA2 levels were associated with worse prognosis, including tumour grade, overall survival, and disease-free survival. This was more prevalent in TNBC subtype compared to other subtypes [112].

### 3.3. EVs Regulating Immune Response in Breast Cancer

Bidirectional communication between tumour cells and the immune system occurs within the TME, with a focus on the role of EVs in releasing immune-associated factors that contribute to the regulation of immune responses in the TME [113]. The EVs are key components in mediating communication between tumour cells and the immune response within the TME [114]. While EVs carry various tumour antigens that could potentially induce immunosuppression, emerging research indicates that tumour-derived EVs play a crucial role in facilitating communication between tumour cells and the immune system [115]. These EVs release immune-associated factors into the TME, influencing the immune response dynamics. Researchers have found that the TNBC had the lowest serum EV levels, while oestrogen receptor (ER)^+^HER2^+^ had the highest. This finding was also reflected in tumour tissues, with ER^+^HER2^+^ tumours showing higher levels of EV markers [116]. The high EV scores in tumours were associated with higher levels of immunosuppressive cells like M2 macrophages and mast cells, and lower levels of tumour-infiltrating lymphocytes (TILs) [116]. TNBC-EVs displayed the most potent immunosuppressive effect, decreasing activated T cells and increasing regulatory T cells and IL-10 production. A study has mentioned EVs derived from different breast cancer cell lines in modulating the TME, specifically focusing on their impact on natural killer (NK) cells and regulatory T cells (T-regs) [117]. EVs from both Luminal B (BT474) and triple-negative (HS578T) breast cancer cell lines cultured in 2D and 3D models triggered activation of CD335^+^/CD11b^+^ NK cells, a subset associated with cytotoxic activity. Interestingly, BT474-derived EVs significantly decreased the population of CD39^+^ T-regs, known for suppressing immune responses [117,118].

### 3.4. EVs and Chemoresistance

**Figure 1:**
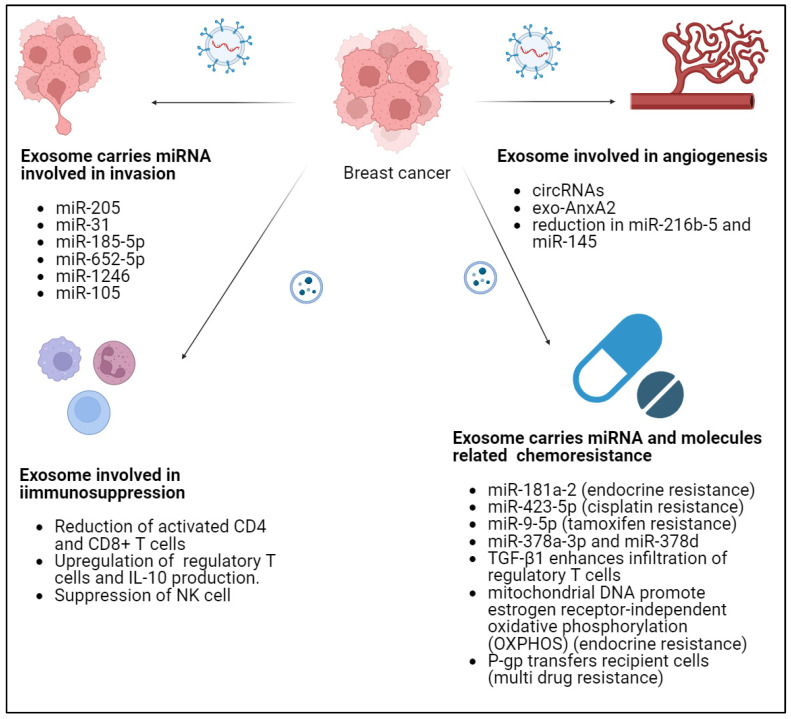
Role of exosome in breast cancer progression.

The emergence of treatment resistance poses a formidable challenge in breast cancer management. Bidirectional communication between breast cancer cells and the TME, facilitated by EVs, plays a pivotal role in treatment response [119]. TNBC, EVs from resistant cells, transfer mitochondria to sensitive cells, heightening resistance. Endocrine therapy resistance in ER^+^ breast cancer involves EVs carrying miRNAs, such as miR-181a-2, disrupting hormonal signalling [120]. Aromatase inhibitor (AI) resistance is linked to elevated EV secretion and Rab GTPase expression [121]. Additionally, resistance to trastuzumab emtansine (T-DM1) in HER2^+^ breast cancer is associated with T-DM1 binding to HER2^+^ breast cancer cell-secreted exosomes [122]. Exosomal miRNAs, including miR-423-5p and miR-9-5p, contribute to resistance against cisplatin and tamoxifen, respectively. Notably, EVs carrying miR-378a-3p and miR-378d activate Wnt and Notch pathways, fostering a drug-resistant phenotype [123]. Meanwhile, development of resistance to radiotherapy due to expression of transforming growth factor-β1 (TGF-β1) is implicated as an endogenous factor, particularly when secreted in an EV-associated form (TGF-β1EV) within radiated tumours which eventually enhanced infiltration of Tregs and phosphorylation of protein kinase C zeta (PKC-ζ) in breast tumour tissue [124]. Another study has demonstrated that EVs released by drug-resistant cancer cells can transfer integral plasma membrane proteins, such as P-glycoprotein (P-gp), to recipient drug-sensitive cells, effectively conferring functional multidrug resistance (MDR) in a short period [125]. The selective packaging of proteins like Ezrin-Radixin-Moesin (ERM) and CD44 in EVs from drug-resistant breast cancer cells has been shown, with this protein complex playing a role in transferring P-gp via EVs and conferring MDR to recipient cells. Moreover, EVs isolated from doxorubicin- and docetaxel-resistant breast cancer cells have been shown to increase drug resistance in non-tumorigenic breast cells by altering the expression of genes associated with cell proliferation and apoptosis pathways [126]. Additionally, EVs released from breast cancer cells can activate signalling pathways like PI3K/Akt, Akt2, FAK, and ERK1/2 in non-tumorigenic breast cells, enhancing their invasiveness and migration abilities [127]. In addition, a study has demonstrated that exosome (Exo)-PD-L1 dose-dependently inhibited the expression of markers of T-cell activation, such as CD3/CD28-driven ERK phosphorylation and NF-κB activation, as well as PHA-induced IL-2 secretion, further showed that Exo-PD-L1 could interact with PD-1 and suppress T-cell cytotoxicity, thereby promoting tumour growth in vivo [128]. Additionally, as the most malignant type of breast cancer, TNBC-derived microparticles can also load PD-L1, especially in patients receiving chemoradiotherapy. Microparticle PD-L1 can negatively regulate CD8^+^ T cells and polarize macrophages to M2, resulting in an immunosuppressive microenvironment that promotes tumour progression [129]. Another study demonstrated that PD-L1^+^ TEVs effectively sequester antibody PD-L1, resulting in accelerated clearance of TEV-bound antibody PD-L1 by macrophages. This leads to an inadequate blockade of tumour PD-L1 and subsequent resistance to antibody PD-L1 therapy [130]. As a result, reduced concentration and faster clearance of the antibodies may result in insufficient time for the drugs to exert their intended action. A study was mentioned that high PD-L2EV levels were associated with reduced progression-free and overall survival. Tumour derived EVs (TEVs) released by tumours may sequester anti-PD-L2 antibodies that are supposed to block immune response against cancer. Decreased PD-L2EV post-chemo is linked to a better response. NOTCH1/ERBB3-positive CTCs and high pre-CT PD-L2EV are associated with shorter progression-free. PD-L2EV emerged as a promising TNBC risk biomarker, possibly guiding anti-PD-1 therapy eligibility [131]. This impairment in the antitumour ability of the immunotherapy could contribute to the development of drug resistance [132]. Horizontal transfer of mitochondrial DNA (mtDNA) in EVs from hormonal therapy-resistant (HTR) metastatic breast cancer patients promotes oestrogen receptor-independent oxidative phosphorylation (OXPHOS) and fosters endocrine therapy resistance in OXPHOS-dependent breast cancer, particularly in cancer stem-like cells [133]. Figure 1 showed the different ways in which exosomes contribute to breast cancer progression. 

## 4. Impact of EV Production in 2D, 3D, and Organoid Culture

The choice between 2D, 3D, and organoid culture systems significantly impacts extracellular vesicle (EV) production and the physiological relevance of cancer models. While 2D cultures offer simplicity and cost-effectiveness, they lack the complexity necessary to replicate the intricate 3D architecture and cell–cell interactions of real tissues, potentially limiting their predictive accuracy for in vivo cellular behaviour [134,135,136,137,138]. In contrast, 3D cultures, including spheroids and organoids, better mimic physiological conditions, promoting increased EV production and offering a more accurate representation of tumour biology [139,140,141,142,143]. Patient-derived xenografts (PDXs) also play a crucial role in preserving the tumour microenvironment and recapitulating intratumor heterogeneity, making them invaluable tools for preclinical drug testing and personalized medicine approaches [144,145,146,147,148,149].

Furthermore, EVs secreted by tumour organoids and patient-derived organoids (PDOs) serve as crucial mediators of intercellular communication, influencing tumour progression and metastasis [150,151,152,153,154,155,156]. PDOs, in particular, offer a robust 3D in vitro model that faithfully mirrors the characteristics of the original tumour, making them valuable predictive biomarkers for treatment response in cancer patients [157,158]. Despite being relatively more costly and time-consuming to establish, PDOs provide a cost-effective platform for high-throughput anti-cancer drug discovery and offer significant potential for advancing personalized medicine and targeted drug development, ultimately contributing to advancements in cancer research and therapy [159]. Table 3 demonstrated overview of the differences between 2D and 3D culture systems for studying EVs.

## 5. Therapeutic Potential of EVs in Breast Cancer

Blood serum miRNAs, which are biomarkers for breast cancer, are enriched in exosomes. Studies have identified specific exosomal miRNAs, such as miR-1246 and miR-21, that are elevated in breast cancer patient plasma, indicating the potential presence of breast cancer [160]. Exosomal miRNAs have also shown promise as biomarkers for treatment response. For instance, plasma-derived exosomal miR-375 and miR-122 were found to predict neoadjuvant response and breast cancer recurrence [161]. Additionally, exosomal miRNAs were associated with various clinical parameters, such as pathologic complete response, in breast cancer patients [162].

Several cancer-related proteins are present in tumour exosomes, and their profiling can potentially predict therapy resistance. Exosomal proteins, such as TRPC5 and GSTP1, have been linked to chemotherapy response and chemoresistance [163]. Furthermore, the levels of certain exosomal proteins, including CD82, were able to distinguish between patients with breast cancer and healthy subjects. Despite the promising potential of exosomes as biomarkers, the field is still in its early stages, and more research is needed to establish standardized procedures for clinical applications [164,165].

Modified exosomes derived from genetically engineered dental pulp mesenchymal stem cells (DPSCs) were used as carriers for delivering the tumour suppressor miR-34a to inhibit the proliferation of breast carcinoma cells [166]. The study also demonstrated that genetically modified DPSCs could secrete exosomes enriched with therapeutic miRNAs, highlighting the feasibility of utilizing exosome-based vehicles for gene delivery [167]. EVs, naturally occurring phospholipid-based particles, showed potential as drug carriers whereby drugs (porphyrins of varying hydrophobicities) were encapsulated into EVs derived from different cell types (endothelial, cancer, and stem cells) through passive and active encapsulation methods (electroporation, saponin, extrusion, and dialysis) [168]. EVs loaded with hydrophilic porphyrins demonstrated a stronger phototoxic effect than free drugs in a breast cancer cell model. These findings provide a solid foundation for developing EVs as effective drug carriers using practical and transferable methods [169]. Moraes et al. observed that human exosomes derived from adipose tissue-MSCs that have been enriched with miR-424-5p would downregulate PD-L1 expression [170]. Tumour cells that received these exosomes presented high levels of apoptosis when co-cultured with T cells. Finally, when miR-424-5p was delivered via exosomes intratumorally in mice, breast cancer grew slowly, and tumour growth was strongly suppressed. Another study has mentioned that EVs, isolated from human umbilical cord mesenchymal stem cells (hUCMSCs) encapsulated with cannabidiol (CBD) using a sonication method, resulted in decreased expression of proteins involved in inflammation and metastasis while increasing the expression of proteins involved in apoptosis. This combination approach has potential clinical significance for reducing side effects and enhancing the therapeutic efficacy of DOX in TNBC [171].

Another study involving SMART-Exos, which are exosomes expressing dual monoclonal antibodies against CD3 for T cells and EGFR for targeting cancer cells, revealed promising results in the context of cancer immunotherapy. SMART-Exos were found to bind to both T cells and EGFR-positive TNBC cells. The in vitro toxicity investigations demonstrated that SMART-Exos induced strong and specific cytotoxicity against TNBC cells expressing EGFR. This suggests that the engineered exosomes have the potential to selectively target and induce cell death in cancer cells, particularly those of the triple-negative breast cancer subtype. These findings highlight the promising role of exosomes, especially those engineered with specific antibodies, in cancer immunotherapy [172,173].

EVs derived from mesothelin (MSLN)-targeted chimeric antigen receptor T cells (CAR-T) cells have demonstrated efficacy in targeting MSLN-positive and triple-negative breast cancer cells. These EVs achieve their antitumour effect by secreting perforin and granzyme B. Notably, the study reported a significant antitumour effect with low toxicity in vivo, utilizing both BT-549 and MDA231-MSLN xenograft breast tumour models. This suggests that EVs derived from CAR-T cells targeting MSLN have the potential to be an effective and low-toxicity treatment strategy for triple-negative breast cancer [174].

A nanoplatform was developed using macrophage-derived exosomes coated on poly(lactic-co-glycolic acid) nanoparticles for targeted chemotherapy of TNBC and to enhance tumour targetability, the exosome surface was further modified with a peptide targeting the mesenchymal-epithelial transition factor (c-Met), which is overexpressed in TNBC cells [175]. The engineered exosome-coated nanoparticles exhibited significantly improved cellular uptake efficiency and antitumour efficacy of doxorubicin. In vivo studies demonstrated remarkable tumour-targeting efficacy, leading to increased inhibition of tumour growth, inducing intense tumour apoptosis. This research suggests that engineered macrophage exosome-coated nanoparticles could be a promising drug delivery strategy for the treatment of TNBC [176].

Another novel strategy involves disrupting exosomes as a means to treat breast cancer. Targeting different steps in the exosomal life cycle, including release, transfer of biomolecules, and uptake, may offer new avenues for managing the disease. Blocking exosome secretion has been shown to increase the sensitivity of cancer cells to chemotherapeutic agents. Additionally, antibodies targeting exosomal surface markers, such as CD9 and CD63, have been demonstrated to stimulate the removal of cancer-derived exosomes by macrophages, reducing metastases in vivo [177].

Clinical trials are underway to evaluate the diagnostic and predictive value of exosomes in breast cancer [178]. A study registered with ClinicalTrials.gov under the identifier NCT05955521, sponsored by Samsung Medical Center, indeed seems to focus on the evaluation of circulating tumour DNA (ctDNA) and exosomes in triple-negative and HER2-positive breast cancer patients who have undergone neoadjuvant chemotherapy. This type of research is crucial for advancing our understanding of the role of exosomes and ctDNA in breast cancer management. Similarly, another clinical trial, NCT05831397, corresponds to a study sponsored by Istituti Clinici Scientifici Maugeri, Italy. The study is focused on EVs in breast cancer patients who have undergone neoadjuvant chemotherapy. Monitoring changes in EVs may offer insights into treatment response, disease progression, and potential biomarkers for breast cancer. This will eventually lead to developing a new method for the detection of tumour-derived-EVs associated proteins is based on the use of Single Molecule Array (SiMoA), a digital ELISA technology able to detect and quantify extremely low concentrations of target proteins or particles. A recruiting study involves the use of GlyExo-Capture technology to isolate glycosylated EV from the serum of both cancer patients and non-cancer individuals. A machine learning technique will be employed to establish an early diagnosis model for breast cancer based on the GlyExo-Capture platform using miRNA sequencing. The findings will then be validated through qPCR experiments [179,180,181,182].

Exosome-mediated RNA interference” or “exosome-based RNAi” refers to the use of exosomes as delivery vehicles for RNAi therapeutics, where the RNA molecules carried by exosomes can silence or regulate gene expression in recipient cells [183,184]. A study investigated targeting the MAPK/ERK pathway in TNBC by silencing MEK1 through RNAi and delivering anti-MEK1 siRNA using exosomes. MEK1 knockdown inhibited MAPK/ERK signalling, reversing the mesenchymal phenotype, reducing cell migration and invasion, and decreasing MMP-2/MMP-9 expression. Exosome-mediated delivery of anti-MEK1 siRNA effectively inhibited tumour growth, metastasis, and angiogenesis, suggesting potential for TNBC therapy [185]. Another study investigated RNA interference (RNAi) targeting the PIK3CA oncogene in breast cancer cells. PIK3CA downregulation via siRNA inhibited the PI3K/AKT/mTOR pathway, reduced cell viability, induced apoptosis, and impaired cell migration, with EMT reversal. Exosome-mediated siPIK3CA delivery showed superior efficacy over lipofectamine-based methods, suggesting exosomes as promising RNAi carriers in breast cancer treatment [186]. Exosomes engineered from HEK293T cells targeted HER2-positive breast cancer cells by expressing a fusion protein of LAMP2b and DARPin G3, enabling specific binding to HER2 receptors. Loaded with siRNA targeting the *TPD52* gene, these engineered exosomes efficiently downregulated TPD52 gene expression in SKBR3 cells by up to 70%. This approach offers potential for gene therapy and drug delivery to HER2-positive cancer cells, enhancing targeted treatment options [187]. Autologous breast cancer cell-derived exosomes exhibit lung-targeting ability, forming CBSA/siS100A4@Exosome nanoparticles. These nanoparticles, combining CBSA and siS100A4, coated with exosome membranes, effectively target the lung, protecting siRNA and demonstrating biocompatibility. In vivo, they outperform CBSA/siS100A4@Liposome, significantly inhibiting breast cancer growth by gene silencing, suggesting promise as postoperative metastasis suppressors [188]. Table 4 explained the potential therapeutic and diagnostic applications of exosomes in breast cancer whereas Table 5 described the summary of clinical trials exploring the role of EVs in breast cancer treatment and diagnosis.

## 6. Challenges and Future Prospective of EVs

One significant challenge in harnessing EVs for drug delivery lies in achieving efficient loading of therapeutic agents. This limitation arises from various factors, including the size and type of the cargo, loading methods, cargo stability, homogeneity of cargo distribution within EVs, the heterogeneity of EV subtypes, and the accurate quantification of loaded cargo [189,190] The diverse nature of EVs, encompassing exosomes, microvesicles, and others, further complicates the optimization process [191]. Researchers are actively working on refining loading protocols, developing engineered EVs with enhanced loading capacities, and exploring different cargo types to overcome these challenges. While achieving high encapsulation efficiencies remains a hurdle, the potential advantages of using EVs, such as their natural targeting abilities and low immunogenicity, continue to drive research efforts in advancing EVs as promising drug delivery vehicles. Exploring new techniques for EV isolation and purification, promising advancements in high-quality storage technologies, clearer understanding and application of EVs, and improved proficiency in nanotechnology are needed [192]. Rapid advancements in advanced EV analytical techniques are providing more information about the biogenesis, content, and function of EVs. High-sensitivity detection of individual vesicles and subpopulations is made possible by flow cytometry techniques including imaging flow cytometry (IFCM) and vesicle flow cytometry (VFC) [193,194]. New developments in electrochemical biosensing, including the integrated magneto-electrochemical sensor (iMEX), offer sensitive and quick exosome detection with potential uses in medical diagnosis [195]. Label-free EV detection and characterisation are possible with surface plasmon resonance (SPR) and quartz crystal microbalance (QCM) technologies [196]. On the other hand, optofluidic smartphone-based devices, such as the mobile exosome detector (μMED), enable a quick and portable diagnosis [197]. Multiplexed assessment of biomarkers on single EVs is made possible by fluorescence-based approaches such as single EV analysis (SEA), which facilitates in-depth characterisation. Paper-based aptasensors and other paper-based platforms provide easy and affordable ways to analyse electric vehicle emissions [198]. These advanced techniques hold great promise for advancing our understanding of EV biology and translating research findings into clinical applications. These developments are expected to provide more practical options for future exosome applications. 

In conclusion, emerging EVs technology holds promise for oncology treatment, but a deeper understanding is essential to navigate the challenges and capitalize on opportunities in the rapidly evolving landscape of cancer nanotechnology and biology.

## Figures and Tables

**Table 1:** Cellular processes in breast cancer progression.

Mechanism	Description	Role in Breast Cancer	Potential Therapeutic Targets
Hypoxia	Activation of cellular transcriptional response mediated by hypoxia-inducible factors (HIFs) under limiting oxygen conditions.	Linked to aggressive phenotype, poor prognosis	HIF inhibitors, FIH inhibitors
Autophagy	Catabolic process involving formation of autophagosomes, essential for cellular homeostasis. Dysfunctional autophagy implicated in tumorigenesis.	Loss of autophagy-related gene signatures in cancer	Beclin1, FOXO transcription factors
Apoptosis	Programmed cell death process. Dysregulation contributes to uncontrolled cell growth and tumour progression.	Evade apoptosis, contribute to tumour growth	p53 activators, Bcl-2 inhibitors, PI3K/AKT inhibitors
Tumour Microenvironment (TME)	Complex ecosystem comprising various cell types, including immune cells, fibroblasts, and endothelial cells. Key role in tumour behaviour and therapy responses.	Shapes tumour progression and therapeutic responses	Immune checkpoint inhibitors, targeting TAMs, anti-angiogenic agents
Cancer Stem Cells (CSCs)	Subpopulation of tumour cells with self-renewal and therapy resistance properties. Interaction with non-malignant cells in the niche promotes tumorigenesis.	Associated with poor prognosis, therapy resistance	Targeting CSC-specific markers, immune checkpoint inhibitors
Angiogenesis	Process of new blood vessel formation. Critical for tumour growth and metastasis.	Essential for tumour progression, metastasis	VEGF inhibitors, angiogenesis inhibitors

**Table 2:** EV Subtypes and their characteristics.

Subtype	Size (nm)	Origin	Release Mechanism	Cargo	Function
Exomeres	<50	Various	ESCRT-independent	Proteins, lipids	Signalling, intercellular communication
Exosomes	30–150	Endosomes	Multivesicular body fusion with plasma membrane	Proteins, mRNA, miRNA, DNA	Signalling, antigen presentation, immune modulation
Microvesicles	100–1000	Plasma membrane	Direct budding	Proteins, lipids, mRNA, miRNA	Cell adhesion, coagulation, inflammation
Apoptotic bodies	1000–5000	Cytoplasm	Membrane blebbing	Cellular debris, DNA, proteins	Clearance of dead cells
Migrasomes	500–3000	Cytoplasm	Dependent on cell migration	Cellular components, small vesicles	Cell migration
Oncosomes	1000–10,000	Tumour cells	Amoeboid protrusions	Tumour proteins, DNA, RNA	Tumour progression, metastasis

**Table 3:** 2D and 3D culture systems for studying EVs.

Aspect	2D Culture	3D Culture
EV Production	Lower production compared to 3D culture	Higher production compared to 2D culture
EV Identity and Purity	Similar identity and purity to 3D culture	Similar identity and purity to 2D culture
Functional Properties	Stronger anti-inflammatory and anti-fibrotic effects compared to 3D EVs	Weaker anti-inflammatory and anti-fibrotic effects compared to 2D EVs
Culture Environment	Cells grown as a monolayer on flat surfaces	Cells grown as spheroids in a more physiologically relevant environment
Impact on EV Composition	Differences observed in protein cargo between EVs from 2D and 3D cultures	Differences observed in protein cargo between EVs from 3D and 2D cultures
Therapeutic Potential	Demonstrated efficacy in mouse model of lung injury	Reduced efficacy in mouse model of lung injury compared to 2D EVs
Research Importance	Highlighted importance of considering culture conditions when studying MSC-derived EVs	Emphasized the impact of culture conditions on EV production and properties

**Table 4:** Exosomes in breast cancer: therapeutic and diagnostic implications.

EV Type	Application	Approach	Examples
Exosomes	Biomarkers for breast cancer	Profiling exosomal miRNAs	miR-1246 and miR-21 identified as elevated in breast cancer patient plasma
		Profiling exosomal proteins	TRPC5, GSTP1, CD82 associated with therapy resistance
	Gene delivery	Modified exosomes as carriers	Genetically engineered DPSC-derived exosomes delivering miR-34a for breast carcinoma inhibition
	Drug delivery	Passive and active encapsulation methods	EVs loaded with hydrophilic porphyrins for enhanced phototoxic effect in breast cancer cells
	Cancer immunotherapy	Engineering exosomes expressing dual monoclonal antibodies	SMART-Exos targeting CD3 and EGFR for specific cytotoxicity against TNBC cells
		CAR-T derived EVs	EVs from CAR-T cells targeting MSLN-positive TNBC cells, secreting perforin and granzyme B
	Diagnostic and predictive value	Profiling exosomal miRNAs	miR-375 and miR-122 predicting neoadjuvant response and breast cancer recurrence
		Profiling exosomal proteins	CD82 levels distinguishing between breast cancer patients and healthy subjects
EVs derived from stem cells	Drug delivery	Modified EVs loaded with therapeutic miRNAs	hUCMSC-derived EVs encapsulated with CBD reducing inflammation and metastasis in TNBC
	Cancer immunotherapy	Modified EVs targeting specific antigens	DPSC-derived EVs enriched with miR-424-5p downregulating PD-L1 expression in tumor cells
EVs derived from macrophages	Targeted chemotherapy	Coating nanoparticles with exosomes	Macrophage-derived EV-coated nanoparticles delivering doxorubicin for TNBC treatment
	Disrupting exosomal life cycle	Blocking exosome secretion	Inhibition of exosome secretion increasing cancer cell sensitivity to chemotherapy
		Antibodies targeting exosomal surface markers	CD9 and CD63 antibodies stimulating removal of cancer-derived exosomes by macrophages

**Table 5:** Summary of clinical trials of EV against breast cancer.

Clinical Trial Identifier	Sponsor	Focus
NCT05955521	Samsung Medical Center	Evaluation of ctDNA and exosomes in triple-negative and HER2-positive breast cancer patients post-neoadjuvant chemotherapy
NCT05831397	Istituti Clinici Scientifici Maugeri, Italy	Study on EVs in breast cancer patients post-neoadjuvant chemotherapy, utilizing Single Molecule Array (SiMoA) technology for protein detection
Recruiting Study	Unknown	Use of GlyExo-Capture technology to isolate glycosylated EVs from serum for early breast cancer detection, validated through qPCR

## Data Availability

No new data were created or analyzed in this study. Data sharing is not applicable to this article.
